# On the Treatment of Piles by Injection of Carbolic Acid

**Published:** 1888-02

**Authors:** Francis L. Haynes

**Affiliations:** Los Angeles, Cal.


### ON THE TREATMENT OF PILES BY INJECTION OF CARBOLIC ACID.

BY FRANCIS L. HAYNES, M. D., LOS ANGELES, CAL.

I have frequently made Allingham's ligature operation. It is easy and effectual, but is followed by retention of urine and great pain, lasting in some cases seven days. One of my cases died from lockjaw, and a similar result followed in a case in the Episcopal Hospital of Philadelphia.

The great objection to this and other operations is that they are operations and involve the use of ether and rest in bed.

Do we possess in carbolic acid injections a safe, speedy and painless cure for internal piles? A brief narrative of some cases so treated will perhaps answer these questions.

### A.—TREATMENT BY WEAK SOLUTIONS.

CASE I.—Wm. C., aged forty-three, having suffered for thirty years, began treatment October 19th, 1882. The tumors were small, hard, and painful, and the rectum protruded to the extent of one inch.

Five minims of the following solution were carefully and slowly injected through a fine, sharp needle into the most prominent pile:

℞. Crystalized carbolic acid........................gr. i.
Glycerine and distilled water of each..........grs. vi.

(See Kelsey, *N. Y. Medical Journal*, Aug. '82, page 131.)

This procedure was repeated three times, at intervals of ten days, and resulted in complete cure. Each injection was followed by moderate pain, lasting two or three minutes.

Five months afterward, he reported that the cure was permanent, though he had been very intemperate, and had suffered both from diarrhea and from constipation.

CASE II.—Annie L., aged forty, multipara, a severe case of ten years' duration. The treatment described under case I was used at four different sittings. Each injection was followed by severe pain, lasting sometimes three days, and by slight sloughing. The result was a temporary cure, but as the cause, constipation, remained, a speedy relapse ensued.

CASE III.—Captain McD., aged fifty, had suffered for twenty years from very large piles which at times bled so profusely as to endanger his life. The treatment described cured him in ten sittings, by producing large sloughs. The pain following each injection was intense, and lasted, on some occasions, two days. By careful treatment, constipation was relieved, and the patient three years afterward had not relapsed.

CASE IV.—John G., aged thirty. Two five-minim injections, of the same solution, resulted in sloughing and cure of two large piles. At the end of three years, as the result of intemperance, hard work and constipation, this man was as bad as ever.

CASE V.—Jane A., aged twenty-five, multipara, suffered severely from piles for two years before beginning treatment.

Sloughing and cure followed two injections, but the pain was intolerable and a crural phlebitis, starting in veins near the sloughing tumors, set in immediately, which kept her in bed for two months and seriously endangered her life.

In these five cases the treatment was the same as that described under case I, except that the intervals between the injections were frequently much longer than ten days.

On reviewing these experiments, I felt great disgust. With one exception, the patient had been cured at the expense of great suffering and, it seemed, some danger to life. Before deciding to abandon injections, it was determined to make a fresh series of experiments, to decide whether the bad results were inherent to the method, or were due to the solution employed.

CASE VI.--Jos. H., aged forty-seven, a wea thy and very intelligent manufacturer.

March 7, 1883. Has had piles for one year. Sometimes they cause great pain and render confinement to bed for several days necessary. Horseback exercise is impossible and carriage riding very difficult. Examination showed two soft, small, pendulous tumors, in a position to be readily nipped by the sphincter. The peremptory orders were, "Cure me, but don't put me in bed a single day!" Injected morphia sulph. gr. 1-12, in ten minims of warm water, into the largest pile. As soon as the tumor presented the slightest evidence of distention, pain became very severe, but soon abated somewhat, and in two hours entirely disappeared. On the 14th, 21st and 28th days of treatment, from five to ten minims of warm water were injected into the tumors with the same result. On the 35th day he stated that the piles had ceased to be painful and that they could be replaced after defecation with less difficulty. It was apparent that they were smaller and harder. This relief may have been partially due to the sharp bleeding which followed each puncture. It seemed probable that injections of water would produce a cure, but that the treatment would be very tedious.

The following solution was now used, five minims (containing gr. $\frac{1}{4}$ crystallized carbolic acid) to each injection:

℞. Crystallized carbolic acid..................
Glycerine, each..........................aagr. i,
Distilled water...........................grs. xviii.

(This solution was used in all the following cases, unless otherwise stated.)

Injections were given on the 35th and 42d days of treatment. Slight smarting followed the injections for twenty-four hours, and for four days some soreness remained. On the 45th day, the first time for fourteen months, the piles failed to protrude during defecation, and he was never troubled with them again. He died one year afterward from apoplexy.

CASE VII.--J. S. H., aged sixty-four, has had severe piles and prolapse of rectum for thirty-seven years. The prostate is greatly enlarged, and straining during urination aggravates his condition. Fifteen injections were made, with the result of producing considerable relief for about one month. The pain after injection was very slight, except when a stale solution was used. On one such occasion inflammation of the tumor ensued and produced severe suffering for one week. Treatment was finally discontinued because the softer portions of the swelling had disappeared and injections into the tough, unyielding mass remaining were very painful and seemed to be without any good result. As in nearly all the cases a minute, punctated slough was noted at the point of inj ction. Had this case not been complicated by a huge prolapse of the rectum, I believe cure would have resulted.

CASE VIII.—Levi R., aged fifty-four. Two piles, each about the size of a walnut, which had existed about one year, were cured by three injections. One of these was attended with considerable pain, but a dose of ten minims was used instead of the usual one of five.

CASE IX.—Tim G., aged forty-nine, bleeding piles size of a goose egg, for five years, cured by eight injections. He complained considerably of pain after each injection, but was never obliged to abstain from his occupation (night-watchman.) The pain was aggravated by attempts to hasten the cure by making two injectiens into different portions of the mass at the same sitting.

CASE X.—John C., aged thirty, a very similar case to the last. A double injection at one sitting produced great soreness, and made it necessary to abstain from treatment for a month. Cured by ten injections.

CASE XI.—Robert C., aged sixty. Duration fourteen years. Two piles, each about the size of a hickory nut, were cured by five injections (at intervals of ten days) and almost without pain.

CASE XII.—Miss R., aged twenty-five, was cured with scarcely any pain, by four injections, of two small piles which had annoyed her greatly for a year.

CASE XIII—Letitia X., aged thirty, suffering from slight prolapse of the rectum and one small pile, which had made her unable to work for nine weeks, and for which a variety of treatment had been used in vain, was cured by one injection.

CASE XIV.—Dr. McC. was cured of two small piles, which had annoyed him considerably for two years, by three injections, at intervals of two weeks. Pain was insignificant and did not interfere with his practice.

CASE XV.—Jos. C., aged thirty, a marked case, duration five years, cured by three injections, at intervals of two weeks, with but slight pain.

I have used this treatment in many other instances, but as I have learned nothing which is not sufficiently detailed already, I do not quote the cases.

To recapitulate:

1. Five minims or less of a solution containing five per cent. of each of pure crystallized carbolic acid and glycerine in distilled water is injected into the softest portion of a pile. The solution should be fresh and colorless; when stale and yellow its use is attended by great pain and inflammation.

2. The injection is made at a point as near the centre of the pile as possible. The needle used should be fine and sharp, and both it and the syringe perfectly clean.

3. The injection causes pain, not generally very severe, and soreness, due to slight inflammation. If more than one injection is made at one sitting, if the solution is stale or is stronger than that given, or if more than five minims are used, undue inflammation and perhaps sloughing ensue.

4. As soon as the mild inflammation due to an injection disappears, but not before, and under no circumstances more frequently than at intervals of ten days, the procedure may be repeated. Slight cases may be cured by from one to four injections ; severe cases may require as manv as thirty.

5. If the case does not improve, *increase* the interval between the injections.

6. Do not attempt to cure hard, semi-cutaneous piles by this method; the distention of almost inextensible tissue is excessively painful.

7. All uncomplicated cases of internal hemorrhoids may be properly treated in this way. Cases of moderate prolapse, secondary to piles, are also thus curable. Hemorrhoids unattended with great dilation of the blood vessels (capillary piles) are frequently cured very rapidly. As may he inferred from the above remarks, the remedy acts by obliterating the blood vessels of the tumor through a slow subacute inflammation.

B.—TREATMENT BY STRONG SOLUTIONS.

The literature of this subject shows such disastrous results from injection of coagulents into vascular tumors, that we may fairly doubt its justifiability. Therefore, I will here merely give one case showing the results of injecting strong carbolic into tumors shut off from the general circulation by means of a constricting wire. But I hesitate to recommend the use of strong solutions, even with this safeguard.

CASE XVI.—Jno. B., aged thirty, large tumors. After twenty-five sittings, in which a five per cent. solution was used, two small but annoying piles remained. It was necessary for the treatment to be hastened, as the patient was obliged to leave the city. One of the piles was seized with rat-toothed forceps, its base constricted by the wire of a nasal snare, until circulation ceased. Then crystallized carbolic acid (liquified by warmth) was injected and the mass turned white. The operation was repeated in two weeks on the second tumor. Result, cure, with but slight inconvenience.

In two similar cases, thus treated, the result was equally satistory.—*Southern California Practitioner.*

